# Tbx2 Terminates Shh/Fgf Signaling in the Developing Mouse Limb Bud by Direct Repression of *Gremlin1*

**DOI:** 10.1371/journal.pgen.1003467

**Published:** 2013-04-25

**Authors:** Henner F. Farin, Timo H-W. Lüdtke, Martina K. Schmidt, Susann Placzko, Karin Schuster-Gossler, Marianne Petry, Vincent M. Christoffels, Andreas Kispert

**Affiliations:** 1 Institute for Molecular Biology, Medizinische Hochschule Hannover, Hannover, Germany; 2 Department of Anatomy, Embryology, and Physiology, Heart Failure Research Center, Academic Medical Center, University of Amsterdam, Amsterdam, The Netherlands; University of Basel, Switzerland

## Abstract

Vertebrate limb outgrowth is driven by a positive feedback loop that involves Sonic hedgehog (Shh) and Gremlin1 (Grem1) in the posterior limb bud mesenchyme and Fibroblast growth factors (Fgfs) in the overlying epithelium. Proper spatio-temporal control of these signaling activities is required to avoid limb malformations such as polydactyly. Here we show that, in *Tbx2*-deficient hindlimbs, Shh/Fgf4 signaling is prolonged, resulting in increased limb bud size and duplication of digit 4. In turn, limb-specific *Tbx2* overexpression leads to premature termination of this signaling loop with smaller limbs and reduced digit number as phenotypic manifestation. We show that Tbx2 directly represses *Grem1* in distal regions of the posterior limb mesenchyme allowing Bone morphogenetic protein (Bmp) signaling to abrogate *Fgf4/9/17* expression in the overlying epithelium. Since *Tbx2* itself is a target of Bmp signaling, our data identify a growth-inhibiting positive feedback loop (Bmp/Tbx2/Grem1). We propose that proliferative expansion of Tbx2-expressing cells mediates self-termination of limb bud outgrowth due to their refractoriness to *Grem1* induction.

## Author Summary

Developmental defects of the limb skeleton, such as variations from the normal number of digits, can result from an abnormal size of the early limb bud. The mechanisms that restrict limb bud growth to avoid polydactyly, i.e. the formation of extra digits, are unclear. Gremlin 1 (Grem1) has been identified as a key regulator in this process via its role as secreted antagonist of Bone morphogenetic protein (Bmp) signaling. But it remains unknown how *Grem1* expression is switched off appropriately to achieve normal limb bud size. Here we show in the mouse embryo that T-box transcription factor 2 (Tbx2) directly represses *Grem1*. We show that Tbx2-positive mesenchymal cells at the posterior margin of the limb bud create a *Grem1*-negative zone that expands concomitantly with limb bud growth. Progressive displacement of the source of Grem1 and its target region, the apical ectodermal ridge, eventually disrupts epithelial-mesenchymal signaling that is crucial for further proliferative expansion. Our data show how local control of signaling activities is translated into the architecture of the adult skeleton, i.e. the number or digits, which helps us to understand the molecular bases of human polydactyly.

## Introduction

Polydactyly, the condition of having more than the normal number of toes and/or fingers is the most frequent form of limb malformation in humans with an incidence of 1∶500. It most commonly occurs as post-axial polydactyly (extra digit(s) towards 5th finger in limb), less common is pre-axial polydactyly (extra digit(s) towards thumb or toe), very rare is central (mesoaxial) polydactyly (with extra digits within the three middle digits). In all cases, the excess digits can be undeveloped and only attached by a little stalk mostly on the small finger side of the hand or fully formed and working. Polydactyly can occur by itself, or more commonly, as one feature of a syndrome of congenital anomalies as e.g. in Pallister-Hall syndrome, Smith-Lemli-Opitz syndrome or Bardet-Biedl syndrome (OMIM 146510, 270400, 209900).

Elucidation of the genetic, molecular and cellular changes that underlie polydactyly (as well as other limb defects) in humans has greatly benefitted from the analysis of normal limb development, and of the consequences of altered gene functions in suitable animal models such as the chicken and the mouse [1]. All of these studies unraveled that proper establishment and elaboration of the two main limb axes during development is crucial for setting up a correct number and identity of digits. In the limb primordium two signaling centers control the morphogenesis along these limb axes. The apical ectodermal ridge (AER), a distal thickening of the ectodermal jacket of the limb bud, controls proximal-distal (from shoulder to finger tips), whereas the zone of polarizing activity (ZPA) in the posterior region of the mesenchymal core mediates anterior-posterior (A-P, from thumb to the small finger) development. Fibroblast growth factors (Fgf4, Fgf8, Fgf9 and Fgf17) and Shh secreted from the AER and ZPA, respectively, specify distal and posterior positional values in the early limb bud mesenchyme (in the mouse until E9.5) [2], [3]. Removal of the signaling centers or the signals, results in time-dependent distal truncations [4] and loss of posterior limb positions (ulna and digits 2–5) [5], respectively. However, both AER-FGFs and ZPA-Shh do not only provide patterning functions, they also account for the massive outgrowth of the limb bud at subsequent stages (from E9.5 to 11.5 in the mouse) by promoting cell survival and proliferative expansion of the distal and posterior limb bud mesenchyme, respectively [6], [7]. During this phase the three primordia of the stylopod (the future upper arm/leg), the zeugopod (lower arm/leg) and the autopod (hand/foot) are laid down and expanded and digit formation is initiated. It has long been noted that AER and ZPA are mutually dependent on each other, thus linking the two signaling centers by an epithelial-mesenchymal (e-m) signaling loop [8], [9], [10]. Fgf signaling is likely to maintain *Shh* directly, whereas Shh signaling to the overlying AER is relayed in the posterior limb bud mesenchyme by Gremlin 1 (Grem1), a secreted antagonist of Bone morphogenetic protein (Bmp) signaling [11], [12], [13]. Inhibition of Bmp signaling allows *Fgf4* expression in the posterior AER enabling the further propagation of the loop. Limb bud outgrowth comes to a halt (around E11.5 to E12.0 in the mouse) when *Fgf4* and *Shh* expression is shut-off due to a rise of Bmp signaling, resulting in cell-cycle exit and initiation of chondrogenic differentiation [10], [14]. Although the precise mechanisms are unclear, refractoriness of ZPA descendants to induce *Grem1* may be of critical importance to self-terminate the e-m signaling loop [15].

*Tbx2* encodes a transcriptional repressor of the T-box gene family that has recently been implicated in digit development. In the mouse, *Tbx2* is expressed in the anterior and posterior mesenchymal flanks of the early limb bud. From E11.5 on, the posterior domain of *Tbx2* extends more distally and is then found in the interdigital mesenchyme (IDM), most prominently in IDM4, and at the distal tips of the digit condensates at E12.5 [16] (Figure S1). Limbs of *Tbx2*-deficient mice exhibit a hindlimb-specific duplication of digit 4 [17]. The spatially restricted nature of this phenotype may be due to redundancy with the closely related *Tbx3* gene. While both genes are coexpressed in the proximal mesenchyme on either limb margin, *Tbx3* is absent from the distal mesenychme of the posterior flank [16] (Figure S1). Exclusive expression of *Tbx3* in the AER may relate to the variable distal truncations and oligodactyly observed in *Tbx3^−/−^* embryos [18]. Retroviral mis- and overexpression experiments in the chick model provided phenotypic outcomes that suggested additional or alternative functions for *Tbx2* (and *Tbx3*) in regulating digit identity rather than digit number [19] and in anterior-posterior positioning of the limb bud [20].

Here, we set out to gain further insight into the role of *Tbx2* in digit development by genetic loss- and gain-of-function experiments in the mouse. We show that increased digit number in *Tbx2*-mutant mice and oligodactyly in embryos overexpressing *Tbx2* (in the limbs) relates to the maintenance of the e-m feedback loop in the posterior limb bud mesenchyme. Tbx2 terminates this loop by locally repressing *Grem1*. Our experiments identify a Bmp/Tbx2/Grem1 loop that counteracts the Shh/Grem1/Fgf4 loop to mediate self-termination of limb bud outgrowth.

## Results

### Etiology of digit 4 duplication in *Tbx2*-mutant hindlimbs

**Figure 1:**
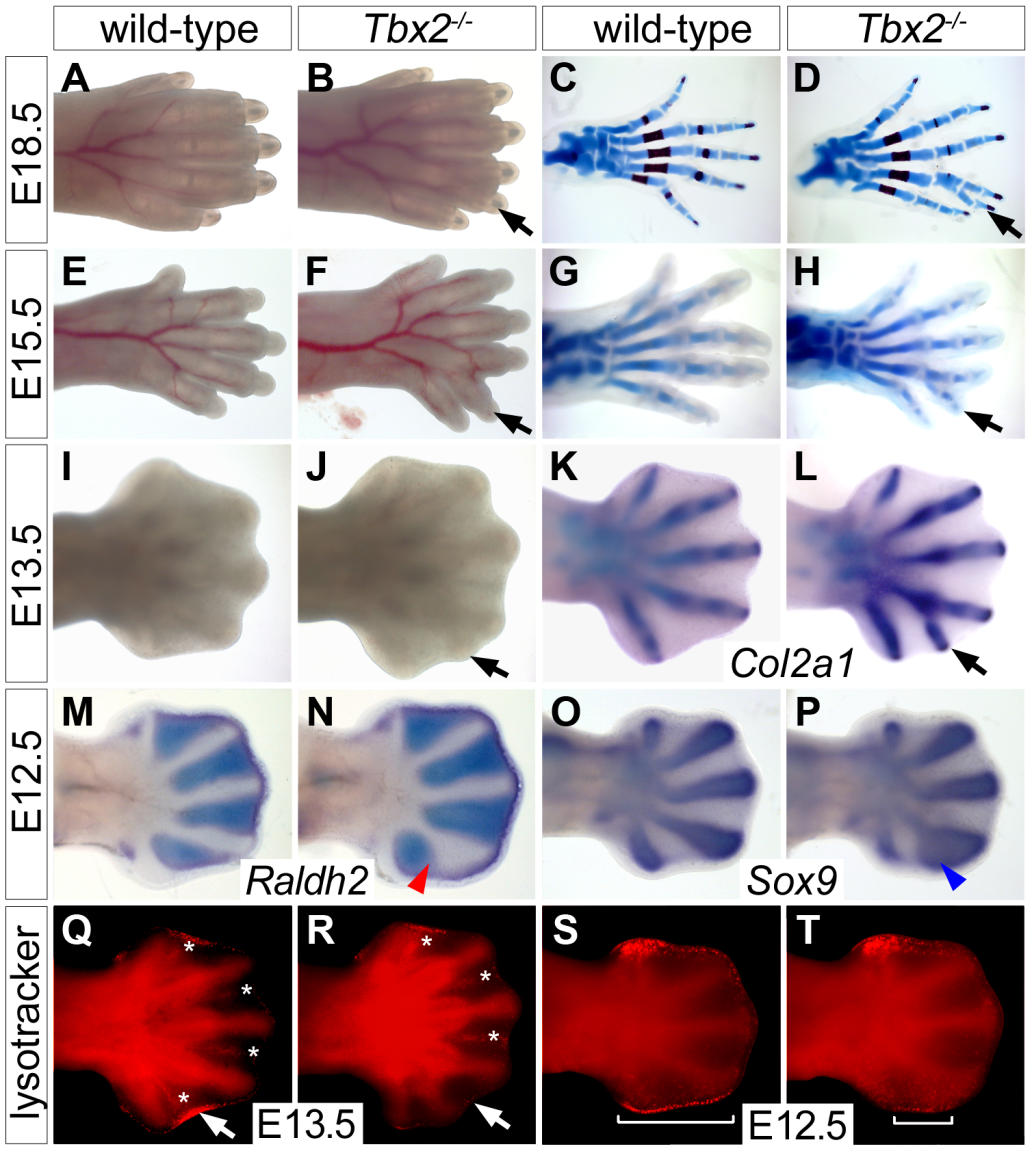
Duplication of digit 4 is preceded by reduced apoptosis and expanded chondrogenesis in the posterior limb mesenchyme. (A–L) Morphology (A, B, E, F, I, J), Alcian blue/Alizarin red skeletal preparations (C, D, G, H) and *Col2a1 in situ* hybridization (K, L) in wild-type and *Tbx2^−/−^* hindlimbs at different stages. Black arrows point to the duplicated digit 4. (M–P) Whole mount *in situ* hybridization analysis for *Raldh2* and *Sox9* expression in E12.5 wild-type and *Tbx2^−/−^* hindlimbs. An expansion of the digit 4 condensation (blue arrowhead) and a reduction of IDM4 (red arrowhead) are observed. (Q–T) Detection of programmed cell death (bright red signals) by Lysotracker Red staining. (Q, R) Asterisks mark interdigital, arrows sub-AER apoptosis. (S, T) Brackets indicate the spatial extent of apoptosis at the posterior limb bud margin.

Mice homozygous for a *Tbx2* null allele (*Tbx2^cre^*) maintained on a NMRI outbred background died shortly after birth due to craniofacial defects [21]. Mutant E18.5 embryos had normal forelimbs but hindlimbs displayed six instead of the normal five digits. Soft tissue webbing, i.e. the persistence of IDM tissue was not observed (Figure 1B). Hindlimb polydactyly showed 70% penetrance in homozygous embryos, heterozygous embryos were not affected. Skeletal preparations of hindlimbs of E18.5 *Tbx2^−/−^* embryos revealed that the proximal segment of digit 4 was broadened and split distally to connect to a duplicated pair of second and third phalangeal segments (Figure 1D). Analysis of chondrogenic elements at E15.5 and E13.5 showed that the duplication of skeletal elements of digit 4 was established shortly after onset of chondrogenesis in the mutant hindlimb (Figure 1H and 1L). Comparative expression analysis for the (pre-)chondrogenic marker gene *Sox9* [22] and *Raldh2*, which marks the IDM [23], showed that the occurrence of duplicated distal cartilagenous segments of digit 4 was preceded by a posterior expansion of the prechondrogenic condensate at the expense of the adjacent IDM4 at E12.5 (Figure 1M–1P).

Since *Tbx2* expression in the developing limb is confined to flank and ID mesenchyme that are removed by programmed cell death [24], we determined the distribution of apoptotic cells by Lysotracker Red staining. We noted a specific reduction of apoptotic cells in IDM4 of *Tbx2^−/−^* hindlimbs at E13.5, whereas other domains of programmed cell death (IDM1-3) were unchanged (Figure 1Q and 1R). In E12.5 wild-type embryos, apoptosis was confined to the mesenchyme underlying the AER at the anterior and posterior margins of the hindlimb (Figure 1S). Programmed cell death appeared unaffected in the anterior region whereas it was largely reduced in the posterior mesenchymal region of mutant hindlimbs at this stage (Figure 1T). The latter may explain the increased size and the characteristic outward curvature of the posterior edge of the mutant hindlimb. Together with the strong expression of *Tbx2* in the distal region of the posterior flank mesenchyme at E11.5 and in IDM4 at E12.5 (Figure S1), this suggests that *Tbx2* functions after E11.5 in the development of the posterior autopod region to maintain IDM4 fate and to restrict the expansion of chondrogenic material at the posterior limit of the digit 4 condensation.

### The signaling loop between the AER and the underlying mesenchyme is disturbed in the posterior region of *Tbx2*-deficient hindlimbs

**Figure 2:**
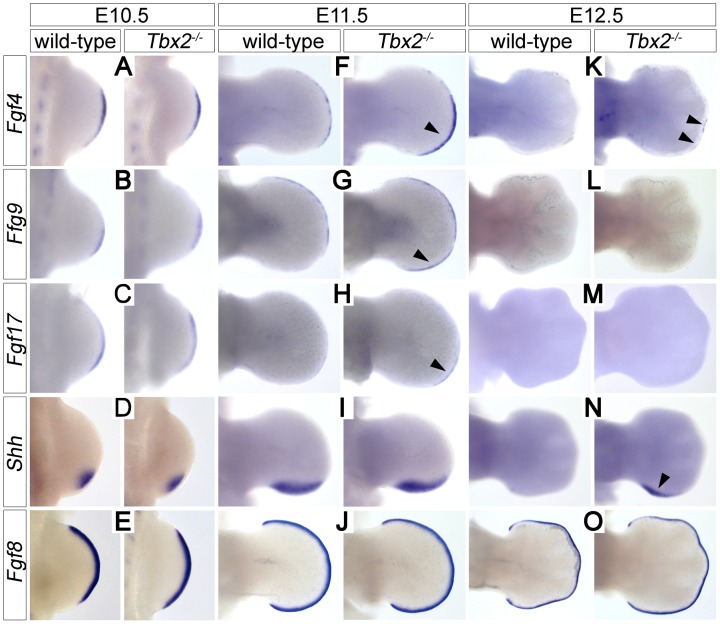
Prolonged posterior e-m signaling in *Tbx2*-deficient hindlimbs. Whole-mount *in situ* hybridization analysis of *Fgf4*, *Fgf9*, *Fgf17*, *Shh* and *Fgf8* expression in E10.5 (A–E), E11.5 (F–J) and E12.5 (K–O) wild-type and *Tbx2^−/−^* hindlimbs. (F, G, H) Arrowheads show prolonged AER expression of *Fgf4*/*9/17* in the posterior limb bud at E11.5. (K, N) Arrowheads show maintenance of *Fgf4* and *Shh* expression at E12.5 in *Tbx2^−/−^* hindlimbs. *Fgf8* expression is unchanged in *Tbx2*-mutant limb buds.

Proliferative expansion and apoptotic removal of the limb bud mesenchyme is governed by reciprocal signaling with the overlying AER [1]. Given the increased posterior size and the decreased apoptosis of E12.5 *Tbx2*-deficient hindlimbs, we studied the expression of factors that are involved in e-m signaling before the manifestation of morphological defects. In E10.5 hindlimb buds expression of *Fgf4*, *Fgf8*, *Fgf9*, *Fgf17* and *Shh* was unaffected (Figure 2A–2E). At E11.5, however, we found increased expression of *Fgf4* (in 7/8 embryos), *Fgf9* (in 3/3 embryos) and *Fgf17* (in 2/3 embryos) in the posterior AER of *Tbx2^−/−^* hindlimbs (Figure 2F–2H). At E12.5, *Fgf4* expression was no longer detected in wild-type hindlimbs, but residual AER expression of *Fgf4* was observed in 3/8 mutant embryos (Figure 2K). *Shh* was unchanged at E11.5 (Figure 2I), but expression was aberrantly maintained at E12.5 (in 5/5 embryos) (Figure 2N). *Fgf8*, which is continuously expressed in the entire AER, was unaffected in *Tbx2*-deficient hindlimbs at all analyzed stages (Figure 2E, 2J and 2O, and data not shown). Together we conclude that Tbx2 is required to assure correct termination of the Fgf4/9/17-Shh signaling loop in the posterior mesenchyme of the hindlimb bud.

### Polydactyly in *Tbx2*-deficient mice is ameliorated by reduced mesenchymal Fgf-signaling

**Figure 3:**
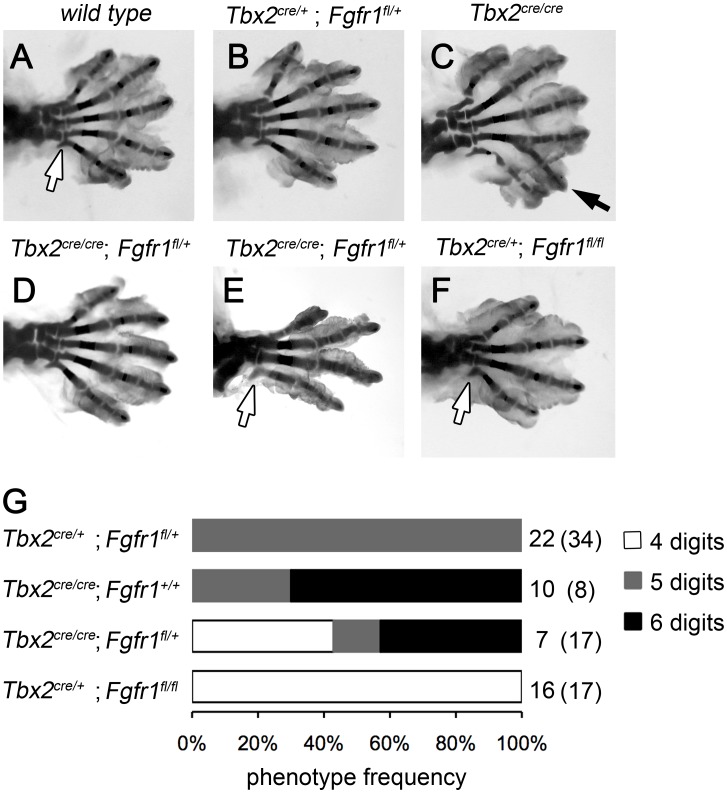
Polydactyly in *Tbx2*-deficient limbs depends on the Fgf signaling level. (A–F) Skeletal analysis of E18.5 hindlimbs. Normal digit development in the wild-type (A) and after heterozygous loss of *Fgfr1* (*Tbx2^cre/+^*;*Fgfr1^fl/+^*, B). Duplicated digit 4 in *Tbx2*-deficient limbs (C, black arrow). Loss of one *Fgfr1* allele on the *Tbx2*-mutant background (*Tbx2^cre/cre^*;*Fgfr1^fl/+^*) rescues polydactyly (D), or even causes oligodactyly (E), as observed in homozygous *Fgfr1*-mutant embryos (*Tbx2^cre/+^*;*Fgfr1^fl/fl^*, F). Morphology of the proximal part of the most posterior metacarpal suggests normal formation of digit 5 (white arrows). (G) Phenotype frequencies in *Tbx2^cre^*/*Fgfr1^fl^* compound mutant limbs (oligodactyly – 4 digits; normal hindlimb – 5 digits; digit 4 duplication – 6 digits). A total of 135 embryos was analyzed. All phenotypes were found in a bilateral symmetric fashion. Numbers on the right side indicate observed and expected counts (in brackets) of embryos for each genotype. Double mutant embryos (*Tbx2^cre/cre^*;*Fgfr1^fl/fl^*) were not obtained at E18.5.

To explore if a gain in e-m signaling indeed causes polydactyly in *Tbx2*-deficient hindlimbs, we genetically reduced the level of mesenchymal Fgf-signaling. We took advantage of cre recombinase driven from our *Tbx2* mutant allele to delete a floxed allele of the *Fgf-receptor 1* gene in the posterior limb bud mesenchyme. Double heterozygous animals (*Tbx2^cre/+^*;*Fgfr1^fl/+^*) were intercrossed and the limb skeleton of compound mutants was analyzed. Homozygous loss of *Fgfr1* (*Tbx2^cre/+^*;*Fgfr1^fl/fl^*) caused a reduced number of 4 digits in fore- and hindlimbs (Figure 3F). This result is consistent with previous experiments using the *Shh-cre* line that recombines in a domain very similar to that of *Tbx2^cre^* [7], [25] (Figure S2A–S2D). At E16.5 *Tbx2^cre/cre^*;*Fgfr1^fl/+^* embryos were underrepresented and *Tbx2^cre/cre^*;*Fgfr1^fl/fl^* embryos were absent, most likely due to a synthetic lethal cardiac defect. Double heterozygous *Tbx2^cre/+^*;*Fgfr1^fl/+^* embryos exhibited a normal limb skeleton (Figure 3B) in agreement with previous results using the *Prrx1-cre* line that deletes in the entire limb bud mesenchyme [25], [26]. In *Tbx2*-deficient embryos, however, dose-reduction of *Fgfr1* strongly reduced the penetrance of polydactyly (3/7 compared to 7/10 in *Tbx2^cre/cre^*;*Fgfr1^wt/wt^* embryos) or even caused oligodactyly (4 digits in 3/7 embryos analyzed) (Figure 3D and 3E). In oligodactic limbs the characteristic shape of the proximal end of the posterior metacarpal indicated a digit 5 identity (white arrows). Absence of *Eomes* expression in E12.5 *Tbx2^cre/cre^*;*Fgfr1^fl/fl^* hindlimbs argued for a specific loss of digit 4 (Figure S2E, S2F) [27]. Our results suggest that polydactyly in *Tbx2*-deficient mice critically depends on increased activity of the e-m signaling loop.

### Tbx2 directly represses *Grem1* in the posterior mesenchyme of hindlimb buds

**Figure 4:**
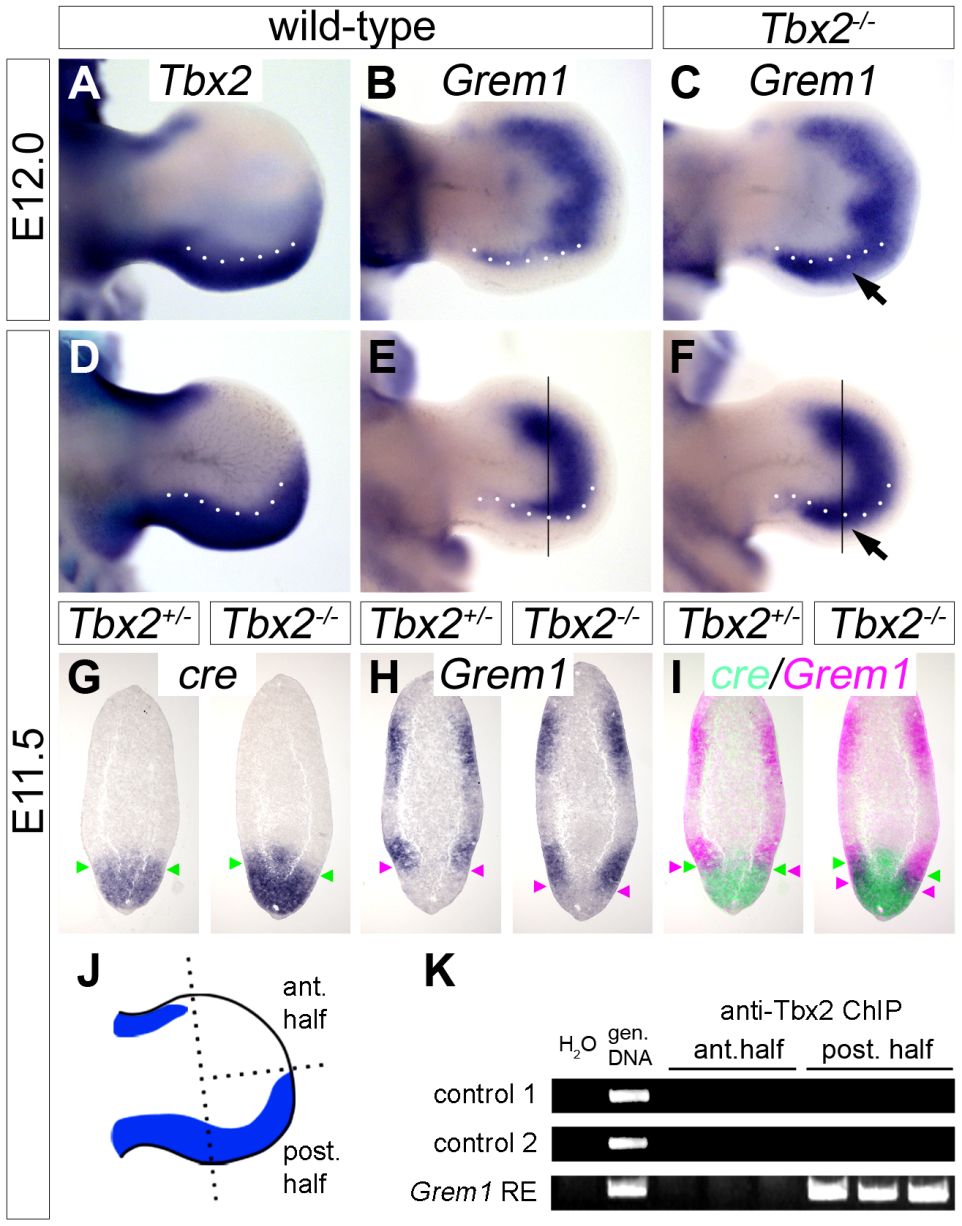
*Tbx2* directly represses *Grem1* expression in the posterior hindlimb mesenchyme. (A–F) Whole mount *in situ* hybridization analysis of *Tbx2* and *Grem1* expression in E12.0 and E11.5 hindlimbs of wild-type and *Tbx2^−/−^* embryos. Arrows (C, F) point to posterior expansion of *Grem1* expression in *Tbx2^−/−^* limbs. White dotted lines demarcate the anterior limit of *Tbx2* expression. (G–I) Comparative expression analysis of *cre*, driven from the mutant *Tbx2*-locus, and *Grem1 by in situ* hybridization on adjacent sagital hindlimb sections of E11.5 *Tbx2^cre/+^* (control) and *Tbx2^cre/cre^* (mutant) embryos. Section planes are indicated by black lines (E, F). Arrowheads mark the anterior limit of *cre* expression (in green), and the posterior limit of *Grem1* expression (in pink). False-colored overlays in (I) show posterior expansion of *Grem1* in *Tbx2^−/−^* hindlimbs. (J–K) ChIP analysis using chromatin from anterior or posterior halves of E11.5 wild-type hindlimbs. Scheme (J) shows *Tbx2* expression (blue) exclusively in the posterior limb half. (K) PCR detection of Tbx2-bound DNA regions. Total genomic DNA was used as positive control. In posterior limb halves Tbx2 binds to the reported *Grem1* limb regulatory element but not to genomic control regions. ChIP experiments were performed in triplicates.

Since *Tbx2* encodes a nuclear transcriptional repressor [28], we sought to identify targets that may help to explain the observed molecular changes. We judged it unlikely, that *Shh* itself is a target of Tbx2 repression since *Tbx2* and *Shh* are broadly coexpressed in the posterior limb bud mesenchyme. Furthermore, unchanged expression of *Shh* in E11.5 mutant hindlimbs cannot explain up-regulation of *Fgf4* in the AER at this stage. Because AER expression of *Fgf4* is mediated by inhibition of Bmp signaling by the secreted Bmp antagonist Grem1 [12], [13], we explored the possibility of *Grem1* regulation by Tbx2. In E12.0 and E11.5 wild-type embryos, the expression of *Grem1* in two crescent-shaped domains at the dorsal and ventral surface of the limb mesenchyme was sharply excluded from the *Tbx2*-positive posterior hindlimb mesenchyme (Figure 4A and 4B, 4D and 4E). In *Tbx2^−/−^* hindlimbs, the expression of *Grem1* was unaffected at E10.5 and E13.0 (data not shown), but appeared specifically up-regulated at E12.0 and E11.5 in the posterior region (Figure 4C and 4F; arrows). On adjacent sagittal sections of E11.5 *Tbx2^cre/+^* hindlimbs, *cre* and *Grem1* were expressed in neighboring, non-overlapping domains (Figure 4G–4I). In *Tbx2^cre/cre^*- embryos, however, *Grem1* expression was shifted posteriorly and overlapped with the domain of *cre* expression that itself was unchanged. Consistent with local expansion of the Bmp-antagonist *Grem1* the Bmp target gene *Dkk1* [29] was absent in the mesenchyme underlying the posterior AER in *Tbx2^−/−^* hindlimbs at E11.5 (Figure S3A). Expression of Bmp targets *Msx1* and *Msx2* [30] was unaffected (Figure S3B and S3C), suggesting that the level of Bmps was still sufficient to activate these genes.

To explore if Tbx2 mediates *Grem1* repression directly, we performed chromatin immunoprecipitation experiments (ChIP) using anti-Tbx2 IgG and E11.5 wild-type hindlimb tissue (Figure 4J and 4K). We found that in posterior limb-bud halves Tbx2 protein specifically interacted with the known *Grem1* limb bud enhancer [31] but not with control genomic regions. No binding was observed to chromatin prepared from anterior limb bud halves that lack *Tbx2* expression, demonstrating specificity of this assay. Together our results strongly suggest that Tbx2 terminates Fgf4/Shh signaling in the posterior limb bud by direct repression of *Grem1*.

### *Tbx2* is a target of Bmp signaling

**Figure 5:**
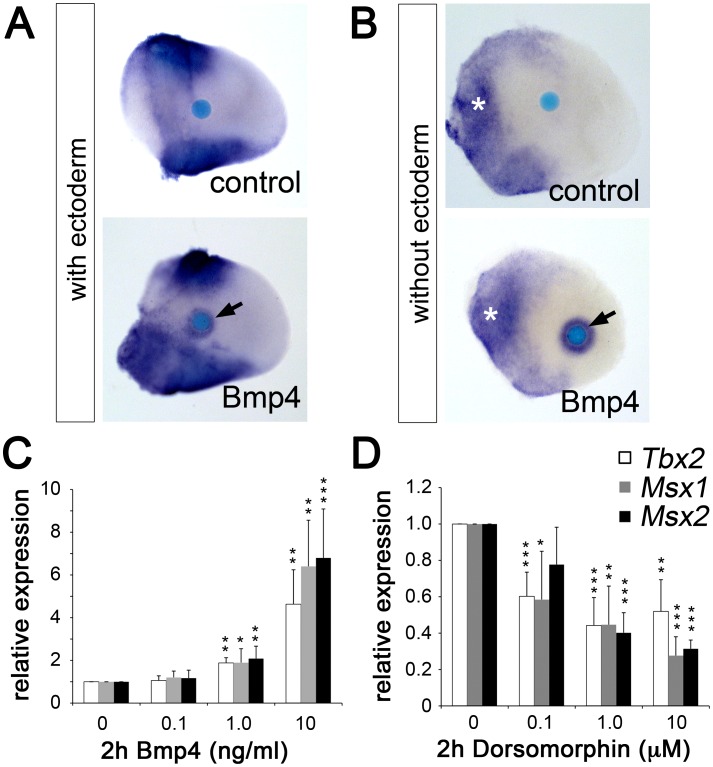
Bmp signaling induces *Tbx2* expression in the limb bud mesenchyme. (A, B) Whole mount *in situ* hybridization analysis demonstrates that Bmp4-soaked beads (100 µg/ml) but not BSA-soaked beads (control) induce *Tbx2* expression in the central limb bud mesenchyme after 18 h of culture (black arrows). White asterisks show an expansion of *Tbx2* expression into the proximal-central region following ectoderm removal. (C, D) Influence of Bmp signaling on *Tbx2* expression in micromass cultures of dissociated E10.5 limb bud mesenchyme. Bmp4 (C) or the Bmp-inhibitor Dorsomorphin (D) was added during the last 2 hours of in total 18 hours of culture. The relative expression levels of *Tbx2* and of the known Bmp targets *Msx1* and *Msx2* were analyzed by qPCR analysis. Means ± SD of four independent cDNAs. Significant changes compared to control are labeled with asterisks (*: p<0.05; **: p<0.01; ***: p<0.001, as determined by t-test).

Bmp ligands are expressed in the AER and at the margins of the limb mesenchyme [13] and represent good candidates as activators of *Tbx2* expression, similar to other developmental contexts [32], [33], [34], [35]. To explore this possibility, we analyzed the effect of Bmp on *Tbx2* expression by bead implantation experiments. We found that Bmp4-soaked beads implanted into the central mesenchyme of E10.5 forelimb buds caused upregulation of *Tbx2* after 16 hours of culture (Figure 5A). This effect was further increased after ectoderm removal (Figure 5B). We established micromass cultures of E10.5 limb bud mesenchyme and studied *Tbx2* expression by quantitative RT-PCR 2 hours after addition of Bmp4 to the medium. A dose-dependent increase of *Tbx2* mRNA was observed that closely resembled induction of the known Bmp targets *Msx1* and *Msx2* (Figure 5C). To study *Bmp* requirement of *Tbx2* expression, we added Dorsomorphin, a selective inhibitor of bone morphogenetic protein (BMP) type I receptors [36], during the last two hours of culture. We observed a twofold reduction of basal *Tbx2* expression in the presence of 1 µM Dorsomorphin that again resembled the response of *Msx1* and *Msx2* (Figure 5D). Thus, Bmp signaling is necessary and sufficient to induce *Tbx2* expression in the limb. Tbx2 – via repression of *Grem1* – therefore operates in a positive feedback loop with Bmp(s) at the posterior margin of the limb.

### Ectopic Tbx2 abrogates *Grem1* expression in the early limb bud mesenchyme

**Figure 6:**
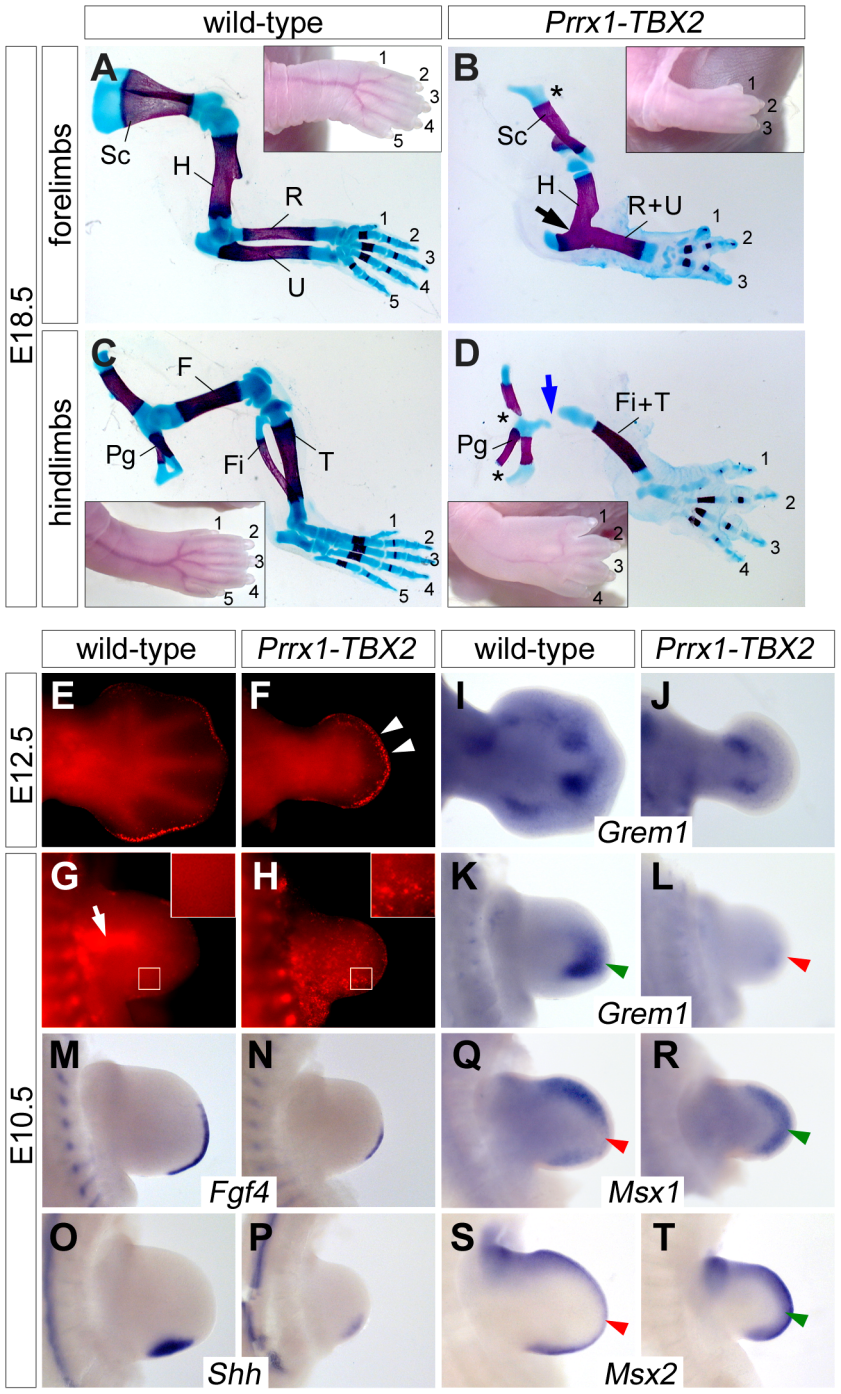
Ectopic Tbx2 represses *Grem1* and disrupts e-m signaling in the limb. (A–D) Skeletal preparations of E18.5 fore- and hindlimbs from wild-type and *Prrx1-TBX2 (Prrx1-cre/+;Hprt^TBX2/Y^)* embryos. Insets show morphology of the autopod. Asterisks indicate reduced scapula in (B), and pelvic girdle in (D). Blue arrow in (D) shows the reduced femur. Abbreviations are: F, femur; Fi, fibula; H, humerus; Pg, pelvic gridle; R, radius; U, ulna; S, scapula; Ti, tibia. (E–H) Detection of apoptosis by Lysotracker Red staining in wild-type and *Prrx1-TBX2* forelimbs. White arrowheads in (F) indicate increased mesenchymal apoptosis beneath the AER. White arrow in (G) shows apoptosis in the proximal mesenchyme of wild-type limbs. White boxes indicate magnified regions. (I–T) Whole-mount *in situ* hybridization analysis of *Grem1*, *Fgf4*, *Shh*, *Msx1* and *Msx2* expression in E12.5 and E10.5 wild-type and *Prrx1-TBX2* forelimb buds. Green and red arrowheads highlight complementary expression of *Grem1* and *Msx1/2*.

Next we used a cre/loxP-based misexpression approach to analyze if expression of *Tbx2* in the entire limb bud would interfere with e-m signaling and digit development. In *Hprt^TBX2^* mice expression of the human *TBX2* cDNA (introduced into the X-chromosomal *Hypoxanthine guanine phosphoribosyl transferase*) locus, is inducible by cre-mediated recombination [37]. We employed the *Prrx1-cre* mouse line to drive transgene expression in the entire limb mesenchyme [38]. X-chromosome inactivation in females causes mosaicism, we therefore only analyzed hemizygous *Prrx1-cre/+*;*Hprt^TBX2/Y^* male embryos that express the transgene in a uniform manner (abbreviated as *Prrx1-TBX2*). By Western blot analysis and immunostainings we confirmed ubiquitous expression of TBX2 in the E10.5 limb mesenchyme at levels comparable to the endogenous Tbx2 protein (Figure S4A–S4D). At E18.5, transgenic embryos exhibited oligodactyly with a dramatic reduction of the limb length (Figure 6A–6D). Forelimbs (3 digits in 9; 4 digits in 1 out of 10 embryos) were stronger affected than hindlimbs (4 digits in 5; 5 digits in 4 and 6 digits in 1 out of 10 embryos), most likely reflecting the relatively delayed onset of *Prrx1-cre* mediated recombination in hindlimbs [38]. We invariantly observed a single zeugopodial element and a reduction of pectoral and pelvic girdles. Forelimb stylo- and zeugopod elements were fused, in the hindlimb the femur was strongly reduced.

The delayed onset of recombination in the hindlimbs led us to study the early consequences of *TBX2* misexpression in forelimbs that demonstrated a complete lack of autopod outgrowth at E12.5. In sharp contrast to the *Tbx2* loss-of-function situation, Lysotracker Red staining showed apoptotic mesenchymal cells underneath the entire AER at this stage in *Prrx1-TBX2* embryos (Figure 6E and 6F). In E10.5 wild-type forelimbs, apoptosis was confined to the proximal mesenchyme as previously reported [6] (Figure 6G). Age matched *Prrx1-TBX2* embryos exhibited strongly reduced forelimb buds and widespread apoptosis throughout the mesenchyme as observed by LysoTracker Red and by TUNEL staining (Figure 6H and Figure S4F).

A highly similar phenotypic spectrum of limb defects including reduced limb length and autopod outgrowth, oligodactyly, fusion of zeugopodial elements, as well as early and widespread mesenchymal apoptosis was reported in *Grem1^−/−^* mutants [12], [13], suggesting that loss of *Grem1* expression may account for the observed effects in *Prrx1-TBX2* embryos. Indeed, we found strong reduction of *Grem1* expression in E10.5 transgenic forelimb buds (Figure 6K and 6L) and residual expression at E12.5 at the base of the autopod (Figure 6I and 6J). Thus, Tbx2 represses *Grem1* expression distally, whereas the more proximal expression domain at E12.5 might be controlled by an independent mechanism. Next, we analyzed the effects of *TBX2* misexpression on known downstream effectors of Grem1. At E10.5, expression of both *Fgf4* and *Shh* was strongly reduced (Figure 6M–6P), again closely resembling the situation in *Grem1^−/−^* limbs. Reduction of the target genes *Spry4* and *Ptch1* [39], [40] confirmed a decrease in Fgf4 and Shh signaling (Figure S5). *Fgf9* and *Fgf17* expression was reduced but expression of *Fgf8* and of the more broadly expressed Fgf target *Etv4* (*Pea3*) [41] was unaffected, demonstrating that Tbx2 acts specifically on posterior e-m signaling (Figure S5C and S5D). To study if reduction of Grem1 is associated with increased Bmp signaling we analyzed *Msx1* and *Msx2* expression, and found that both genes were upregulated in the distal limb bud (Figure 5Q–5T), i.e. in regions normally devoid of *Msx1/2* expression due to Grem1-mediated Bmp-antagonism (compare Figure 5K) [12], [13].

Notably, we did neither observe transformations of digit identity nor changes in limb positioning (data not shown), as reported for *Tbx2* and *Tbx3* misexpression in the chick model [19], [20]. Hence, control of digit formation by local repression of *Grem1* is the primary function of Tbx2 in the mouse.

## Discussion

Precise termination of the e-m signaling loop involving Shh, Grem1 and Fgfs is crucial to restrict limb bud size and to assure a normal digit number. Studies in chick and mouse have indicated that downregulation of *Grem1* drives termination of this loop but suggested two different molecular mechanisms: The one is based on the observation that Shh expressing cells as well as their descendants are unable to express *Grem1* [15], [42]. Proliferative expansion of ZPA-derived cells [7] would thereby displace the source of Grem1 secretion from the AER to a point where the distal range of Grem1 diffusion is eventually exceeded. As a consequence, Bmp signaling increases and suppresses AER-*Fgfs*, followed by termination of *Shh* expression and proliferative expansion. Although this model is supported by the sequence of signal terminations in the chick, the factor responsible for the cell-autonomous repression of *Grem1* in the Shh lineage cells has remained enigmatic (Scherz et al., 2004). As an alternative mechanism, recent loss-of-function experiments in the mouse have supported the existence of an inhibitory Fgf/Grem1 signaling loop that becomes progressively activated once the positive Shh/Grem1/Fgf loop has induced sufficiently high levels of Fgfs [43]. This model can elegantly rationalize the regulation of limb bud size via interconnected, self-terminating signaling loops. However, it fails to explain the selective absence of *Grem1* from the posterior limb bud mesenchyme.

**Figure 7:**
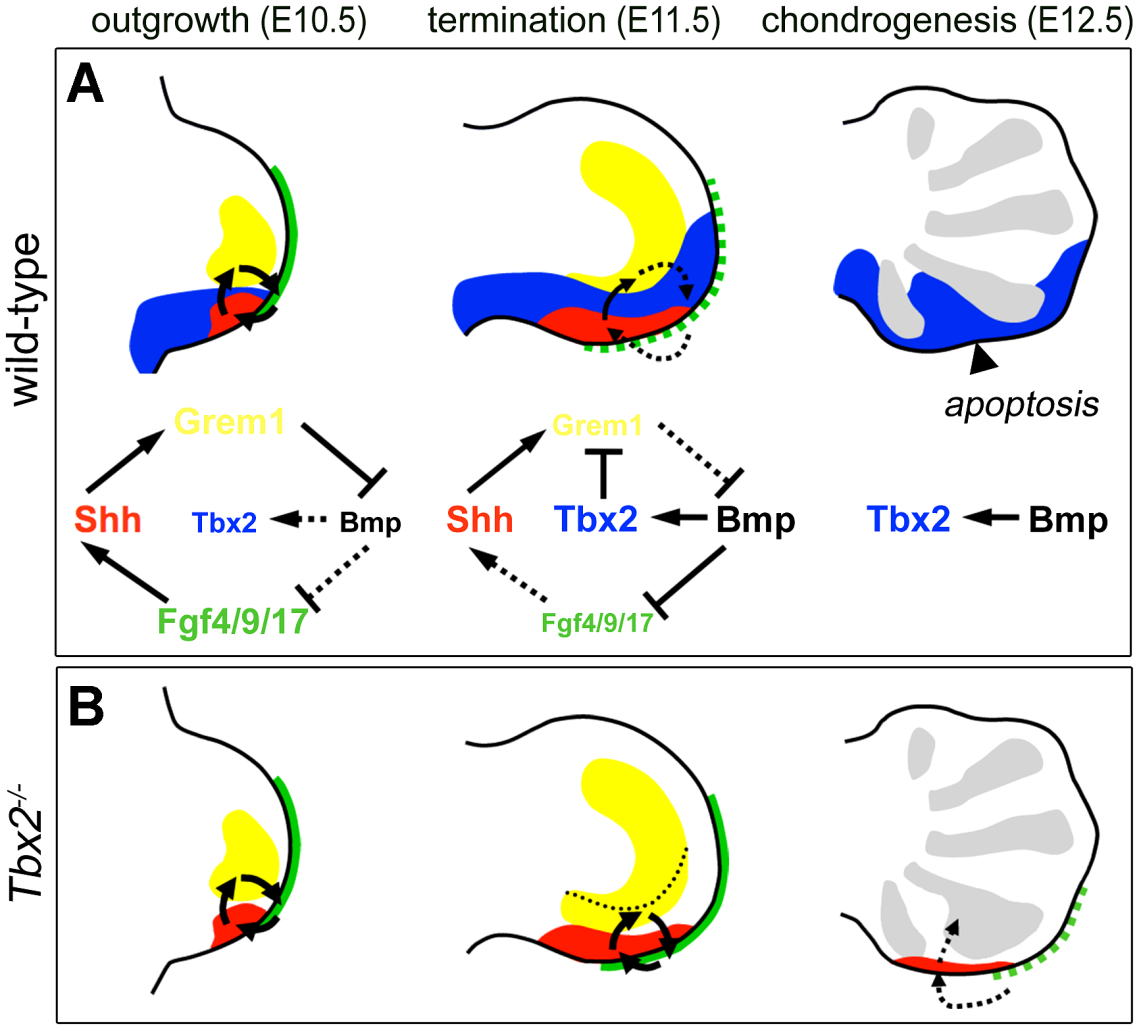
Model of Tbx2 function in the posterior hindlimb mesenchyme. (A) During normal limb bud outgrowth (E10.5) the expression domains of *Shh* (red), *Grem1* (yellow) and *Fgf4/9/17* (green) are in close proximity allowing propagation of the e-m signaling loop. Following proliferative expansion of ZPA-derived cells, a broadened *Tbx2* expression (blue) causes *Grem1* repression. Progressive displacement of Grem1-secreting cells from the AER terminates e-m signaling (E11.5, dotted arrows). The diagrams illustrate these dynamic signaling activities. (B) Failure of *Grem1* repression in *Tbx2*-deficient hindlimbs causes prolonged e-m signaling. The dotted line shows the posterior limit of *Grem1* expression in the wild-type. Impaired termination of outgrowth reduces apoptosis and causes expansion of the digit4 condensation (E12.5, grey regions).

The parallel upregulation and prolonged expression of *Grem1*, *Fgf4/9/17* and *Shh* in *Tbx2*-deficient hindlimbs and their coordinated downregulation upon limb-specific *TBX2* overexpression identifies Tbx2 as an essential factor for the termination of the e-m signaling loop (summarized in Figure 7). Given the virtual overlap of Tbx2 and Shh cell lineages (see Figure S2A–S2D and [7]), we propose that Tbx2 renders Shh-descendant cells unable to induce *Grem1*. Consistently we found that the transcriptional repressor Tbx2 binds to the *Grem1* limb enhancer *in vivo*. This 437 bp element has been identified previously by genome-wide, limb-specific ChIP analysis of Gli3, the transcription factor that mediates Shh-dependent gene transcription in the limb bud [31]. In transgenic mice the element largely recapitulates the complex *Grem1* limb expression pattern, which argues for an integration of both activating (Gli3) as well as repressive modules (Tbx2). Absence of *Tbx2* expression from the anterior limb margin can explain earlier observations that Shh loaded beads are sufficient to induce *Grem1* in this region but not in the posterior limb mesenchyme [15]. Importantly, we noted that in *Tbx2*-deficient hindlimbs the posterior mesenchyme directly underneath the AER remained *Grem1* negative (Figure 4F). This suggests that the negative Fgf-Grem1 signaling loop [43] stays active in *Tbx2^−/−^* embryos and argues that both termination mechanisms (see above) operate in parallel in adjacent domains of the limb mesenchyme to achieve spatio-temporal control of *Grem1* expression.

The hindlimb-specific requirement for *Tbx2* cannot easily be explained at this point. It may result from differences in the proliferative expansion of fore- and hindlimbs, differential Shh/Fgf signaling activities, or the existence of additional repressors that might operate redundantly with Tbx2 in forelimbs. Tbx3 is unlikely to compensate for the loss of Tbx2 given the absence of *Tbx3* expression at the distal margin of forelimb buds (Figure S1). However, we can clearly exclude a function of mouse *Tbx2* in specification of digit identity, as previously suggested from experiments in chicken embryos [19]. Although some of the observed differences may relate to species-specific variations in the underlying molecular programs, experimental caveats due to unphysiological levels of protein obtained after retroviral mis- and overexpression cannot be excluded at this point.

We have shown that Bmp signaling activates *Tbx2* expression in the limb mesenchyme. This notion is supported by reduction of *Tbx2* expression in embryos with reduced levels of Bmp signaling in the limb [44]. Since *Tbx2* expression is normal in *Prrx1-cre*;*Bmp2^fl/fl^*;*Bmp4^fl/fl^* or *Prrx1-cre*;*Bmp2^fl/fl^*;*Bmp7^−/−^* mice [45], several Bmp ligands are likely to operate redundantly. Recent transgenic analysis of the *Tbx2* promoter led to the identification of Smad binding sites [34], [35] that mediate *Tbx2* expression in the limb (supporting material in [35]) further stressing the relevance of Bmp signaling for *Tbx2* expression in this organ. In chick embryos the non-AER marginal ectoderm activates *Tbx2* expression, whereas transplantation of the AER or Fgf-soaked beads repress *Tbx2* expression [45]. In contrast to chick embryos, however, murine *Tbx2* expression is not excluded from mesenchymal regions underling the AER and extends more distally, demonstrating species-specific differences in *Tbx2* regulation. Abrogation of Shh signaling by cyclopamine treatment has shown that Shh is dispensable for posterior *Tbx2* expression [45]. The fact that implantation of Shh-soaked beads can induce *Tbx2* expression in the anterior mesenchyme [46] may therefore be secondary to induction of Bmp expression in these regions [42]. Thus, Bmp signaling constitutes a major positive input for *Tbx2* limb expression but additional factors may feed-in to fine-tune its expression. However, the mutual relationship: Bmp-induction of *Tbx2* and Tbx2 repression of Grem1-mediated Bmp-antagonism, constitute a positive feedback loop that acts locally to diminish and terminate Shh/Fgf4 signaling (Figure 7). Postaxial polydactyly, expansion of *Sox9* expression and reduced apoptosis in Bmp pathway mutants [44], [47] represent phenotypic similarities that support engagement in a common pathway.

While loss of *Grem1* (following *TBX2* misexpression) causes a general increase in Bmp signaling, as indicated by induction of distal *Msx1/2* expression, both genes were unaffected in *Tbx2^−/−^* limbs. Here, absence of the Bmp target *Dkk1* was observed in the sub-AER mesenchyme, indicating that *Dkk1* expression requires higher levels of Bmp signaling. In fact, the loss of *Dkk1* expression might explain reduced apoptosis in the *Tbx2^−/−^* sub-AER mesenchyme as ectopic Dkk1 induces programmed cell death [29]. Moreover, the postaxial polydactyly in *Dkk1* mutants [48] is compatible with a role downstream of *Tbx2*.

Mesoaxial polydactyly has been suggested as a characteristic feature of Oral-Facial-Digital syndrome (OFDS) type IV (OMIM 258860) [49]. Interestingly, a variant case of OFDS, that partially resembles OFDS type IV and type II (also known as Mohr Syndrome, OMIM 252100) shows a malformation spectrum including endocardial cushion defects, cleft palate and central polydactyly with bifurcated Y-shaped metacarpals of the forth digit [50] phenocopying *Tbx2* loss-of-function in the mouse [17], [21], [51].

Together, our data allow the integration and refinement of existing models for termination of distal limb outgrowth, and emphasizes how local differences of signaling activities are translated into the architecture of the adult skeleton, i.e. the number or digits. They show that central polydactyly like preaxial and postaxial variants arise from perturbation in components of the signaling loops including Shh, Grem, Bmp and Fgf signaling.

## Materials and Methods

### Ethics statement

All animal work conducted for this study was approved by H. Hedrich, state head of the animal facility at Medizinische Hochschule Hannover and performed according to German legislation.

### Mice and genonotyping

Mice carrying a null allele of *Tbx2* (*Tbx2^tm1.1(cre)Vmc^*, synonyms: *Tbx2^−^, Tbx2^cre^*) [52], a floxed allele of *Fgfr1* [53], the transgenic lines *Tg(Prrx1-cre)1Cjt/J*) (synonym: *Prx1-Cre*) [38] and the reporter lines *R26^lacZ^* (synonym: *R26R*) [54] and *Tg(CAG-Bgeo/GFP)21Lbe)* (synonym: *Z/EG*) [55] and mice with integration of the human *TBX2* gene in the *Hprt* locus (*Hprt^tm2(CAG-TBX2,-EGFP)Akis^*, synonym: *Hprt^TBX2^*) [51] were maintained on an outbred (NMRI) background. For timed pregnancies, vaginal plugs were checked in the morning after mating; noon was taken as embryonic day (E) 0.5. Pregnant females were sacrificed by cervical dislocation; embryos were harvested in phosphate-buffered saline, decapitated, fixed in 4% paraformaldehyde overnight, and stored in 100% methanol at −20°C before further use. Genomic DNA prepared from yolk sacs or tail biopsies was used for genotyping by polymerase chain reaction (PCR).

### Limb culture experiments

Limb buds from E10.5 wild-type NMRI embryos were dissected in PBS and placed on Nucleopore filters (Whatman, pore size 1.0 µm) on top of a stainless steel mesh at the air-liquid interface in 3.5 cm cell culture dishes. The surgical removal of the ectoderm was performed with forceps in DMEM/10% FCS, after incubation of limb buds in 2% Trypsin/PBS (w/v) for 20 min at 4°C. Affi-Gel blue beads (100–200 µm diameter, Bio-Rad) were rinsed in PBS and incubated at room temperature for 1 h in either recombinant human BMP4 (100 µg/ml, AbD Serotech) or in 1 mg/ml BSA (control). Beads were rinsed in PBS before implantation into the limb mesenchyme. The culture was performed at 37°C and 5% CO_2_ in organ culture medium (DMEM/10% FCS, 1× solutions of Penicillin/Streptomycin, Glutamax, sodium pyruvate, and non-essential amino acids [Gibco]).

### Limb micromass cultures

Micromass cultures were established by dissociation of E10.5 fore- and hindlimb buds in DMEM/10% FCS, after incubation in 2% Trypsin/PBS (w/v) for 5 min at 37°C. A single cell suspension was obtained by gentle pipetting; clumps of ectoderm were removed after sedimentation. Cells were adjusted to 1.5×10^7^ cells/ml in organ culture medium (as above), before 10 µl spots were placed on 24 well plates. Cells were incubated for 1 hour at 37°C to allow adherence, before the wells were filled with medium. Recombinant BMP4 (as above) or Dorsomorphin (Sigma) were added to the medium after 16 h of culture, 2 hours before RNA isolation. Total RNA was extracted from single micromass cultures with PeqGOLD reagent (Peqlab). RNA (500 ng) was reverse transcribed using oligo dT primer and RevertAid M-MuLV Reverse Transcriptase (Fermentas) following the manufacturer's recommendations. Relative gene expression was measured using iQ^−^SYBR Green reagent (Biorad) and calculated using the DDCT method by normalization to *Hprt* expression. The error bars show standard deviation from 4 independent experiments.

### *In situ* hybridization, skeletal Preparations, β-galactosidase and apoptosis assays, and immunostainings

Skeletal preparations with Alcian blue and Alizarin red, β-galactosidase stainings, detection of apoptosis by the TUNEL assay, and *in situ* hybridization analyses on whole embryos and on 10 µm paraffin sections were performed as previously described [56], [57], [58]. All experiments were performed on at least three independent embryos. For experiments that showed variable results, numbers of used specimens are mentioned in the text. For detection of apoptotic cells, embryos were collected in PBS and incubated for 30 min at 37°C in prewarmed PBS containing 2.5 µM LysoTracker Red (Sigma), followed by several washes in PBS. Tbx2 antibody (#07-318, Millipore) was used 1∶100 for immunostainings on 5 µm paraffin sections. Signals were amplified by Tyramide Signal Amplification (PerkinElmer).

### Chromatin immunoprecipitation

Distal pieces of E11.5 wild-type hindlimb buds were separated in anterior and posterior halves (as indicated in Figure 4J) and collected in 3 separate pools of 8 embryos each and treated with 4% paraformaldehyde overnight. ChIP experiments using an anti-Tbx2 antibody were performed essentially as previously described [59]. Primer sequences for the *Grem1* enhancer were TTCCCCTCCTCTTCCACAGTAGG and GGCCAAATAACCACACAGGAAAC, corresponding to a 447 bp fragment previously tested in transgenic animals [31]. Primer pairs of control regions were TGAAAACCCCAAGGAGTCTG, CATGGGCAGGATACTACGCT (193 bp product, 25.5 kbp distal) and AGCCTGACTCTCCCATCTCA, GGCACTGGATAAAACTCCCA (273 bp product, 24.3 kbp distal from the *Grem1* limb enhancer).

### Image analysis

Whole-mount specimens were photographed on Leica M420 with Fujix digital camera HC-300Z. Whole-mount GFP-epifluorescence was documented on a Leica MZFLIII macroscope equipped with a Leica DFC300 camera. Sections of *in situ* hybridizations were photographed using a Leica DM5000 microscope with a Leica DFC300FX camera. All images were processed in Adobe Photoshop CS.

## Supporting Information

Figure S1 *Tbx2* and *Tbx3* exhibit dynamic expression in the developing mouse limb bud. Comparative expression analysis of *Tbx2* and *Tbx3* in whole forelimb (A–F) and hindlimb buds (G–L) of wild-type mouse embryos by *in situ* hybridization. Probes used and embryonic stages are indicated in the figure. At the posterior limb bud margin the distal limit of expression is highlighted by blue arrowheads, demonstrating that *Tbx2* expression extends further distally compared to *Tbx3*. The expression of *Tbx3* in the AER is indicated by black arrowheads. At E12.5, *Tbx2* shows strong expression in the IDM4 (asterisks). Note that the murine expression patterns of *Tbx2* and *Tbx3* expression were inverted in a previous publication [19].
(TIF)

Figure S2 (A–D) *Tbx2* expressing cells contribute to posterior digits 3, 4 and 5. (A, B) X-Gal stainings to detect ß-galactosidase activity in E13.0 *Tbx2^cre/+^*;*R26^lacZ/+^* fore- and hindlimbs. Cells previously expressing *Tbx2* as detected by β-galactosidase activity are present in the anterior and posterior flank mesenchyme and in the posterior half of the autopod encompassing the anlagen of digit 4 and 5, and partially of digit 3. (C, D) GFP-epifluorescence analysis in E18.5 *Tbx2^cre/+^*;*ZEG^GFP/+^* embryos detects the final contribution of the *Tbx2-cre* ^+^ cell lineage to fore- and hindlimbs. Digit 3 is partially, digits 4 and 5 are completely derived from *Tbx2* expressing cells. Note that the *Tbx2*-positive domains in the anterior and posterior flank mesenchyme do not substantially contribute to the E18.5 limb since they are most likely removed by apoptosis during development. (E, F) *In situ* hybridization analysis of *Eomes* expression as marker for digit 4 identity. At E12.5 wild-type hindlimb buds show a proximal domain of *Eomes* expression (arrow) that indicates formation of digit 4. Absence of signal in *Tbx2^cre/cre^*;*Fgfr1^fl/fl^* hindlimbs argued for a specific loss of digit 4 in oligodactic individuals.
(TIF)

Figure S3 (A–C) Expression analysis of the Bmp target genes *Dkk1*, *Msx1* and *Msx2* by whole mount in situ hybridization in E11.5 wild-type and *Tbx2^−/−^* hindlimbs. Magnified regions (1 and 2) in (A) show loss of mesenchymal *Dkk1* expression in the posterior limb bud region (arrow) of *Tbx2^−/−^* mutants but maintained expression in the adjacent AER (asterisks). (B–C) Unaffected expression of *Msx1* and *Msx2*.
(TIF)

Figure S4 TBX2 protein levels and apoptosis in misexpression embryos. (A, B) Analysis of endogenous Tbx2 and transgenic TBX2 protein expression. Anterior and posterior halves of E11.5 forelimbs were collected (as shown in the scheme in A), and lysates were analyzed by Western blot. TBX2 misexpression in *Prrx1-TBX2 (Prrx1-cre/+;Hprt^TBX2/Y^)* embryos was found at physiological levels. β-actin Western blot is shown as a loading control. (C, D) Immunostaining of endogenous and ectopic Tbx2 expression (red signal) in control and *Prrx1-TBX2* embryos. Sagittal E10.5 forelimb sections are shown. Posterior restriction of Tbx2 in the wild-type (C) and ubiquitous mesenchymal expression of transgenic TBX2 protein (D). Same Tbx2 antiserum used as in (B). (E, F) TUNEL staining on sagittal E10.5 forelimb sections shows widespread mesenchymal apoptosis in *Prrx1-TBX2* embryos.
(TIF)

Figure S5 Selective disruption of posterior e-m signaling following *TBX2* misexpression in the limb. (A–F) *In situ* hybridization analysis of *Spry4*, *Ptch1*, *Fgf9*, *Fgf17*, *Fgf8* and *Etv4* expression in E10.5 wild-type and *Prrx1-TBX2 (Prrx1-cre/+;Hprt^TBX2/Y^)* forelimbs. *Spry4*, *Ptch1*, *Fgf9* and *Fgf17* are strongly reduced (red arrowheads) following *TBX2* misexpression, whereas the levels of *Fgf8* and *Etv4* are not strongly affected. Note that in wild-type limb buds the expression of *Spry4* is more restricted to the posterior-distal mesenchyme as compared to *Etv4* that is expressed beneath the entire AER.
(TIF)
