# Supplementary figures and images for: Tbx2 Terminates Shh/Fgf Signaling in the Developing Mouse Limb Bud by Direct Repression of Gremlin1
Source: PLoS Genet. 2013 Apr 25;9(4):e1003467. doi: 10.1371/journal.pgen.1003467 (PMC3636256; doi:10.1371/journal.pgen.1003467)

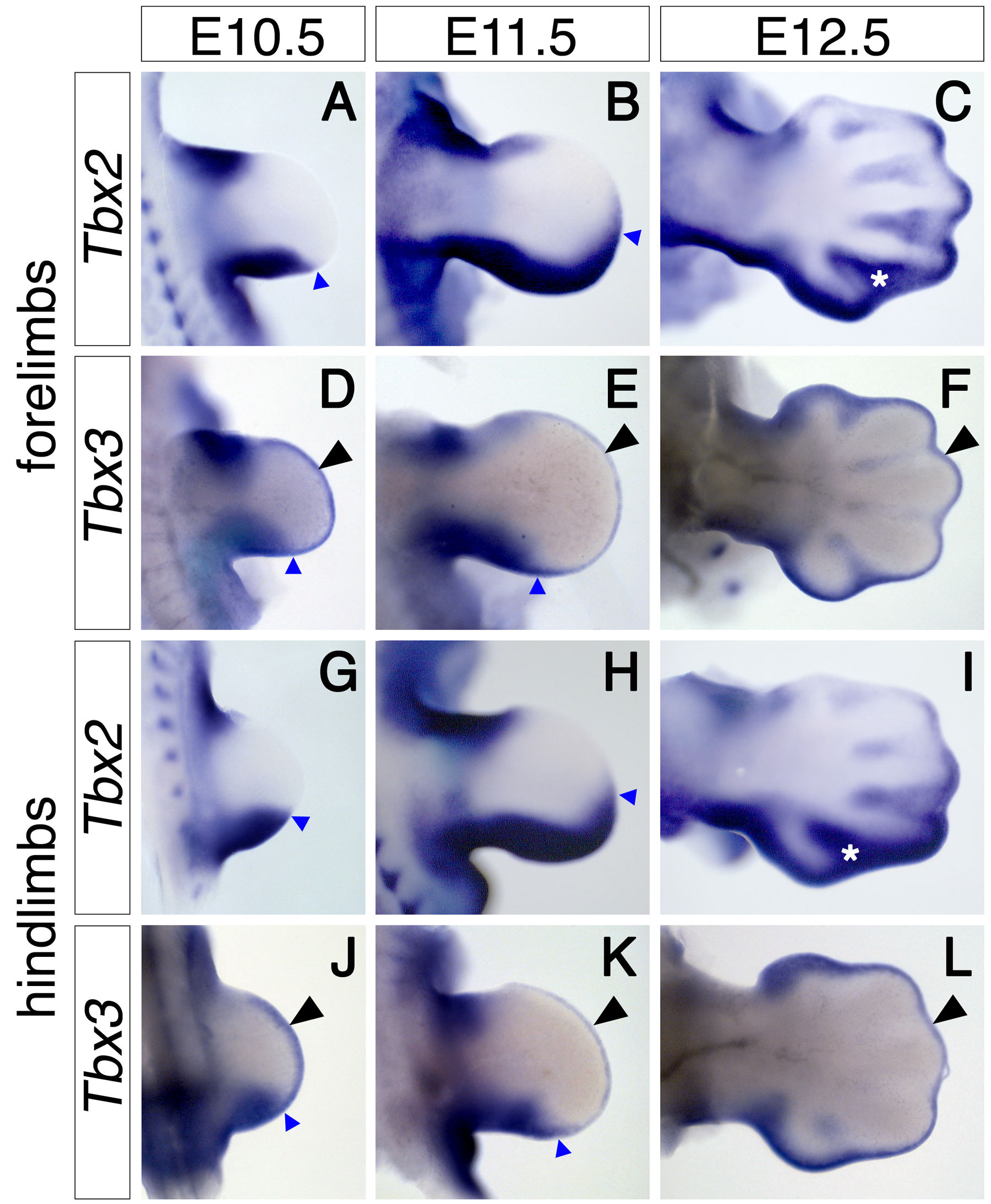

Supplement: Figure S1 — Tbx2 and Tbx3 exhibit dynamic expression in the developing mouse limb bud. Comparative expression analysis of Tbx2 and Tbx3 in whole forelimb (A–F) and hindlimb buds (G–L) of wild-type mouse embryos by in situ hybridization. Probes used and embryonic stages are indicated in the figure. At the posterior limb bud margin the distal limit of expression is highlighted by blue arrowheads, demonstrating that Tbx2 expression extends further distally compared to Tbx3. The expression of Tbx3 in the AER is indicated by black arrowheads. At E12.5, Tbx2 shows strong expression in the IDM4 (asterisks). Note that the murine expression patterns of Tbx2 and Tbx3 expression were inverted in a previous publication [19]. (TIF) [file pgen.1003467.s001.tif]

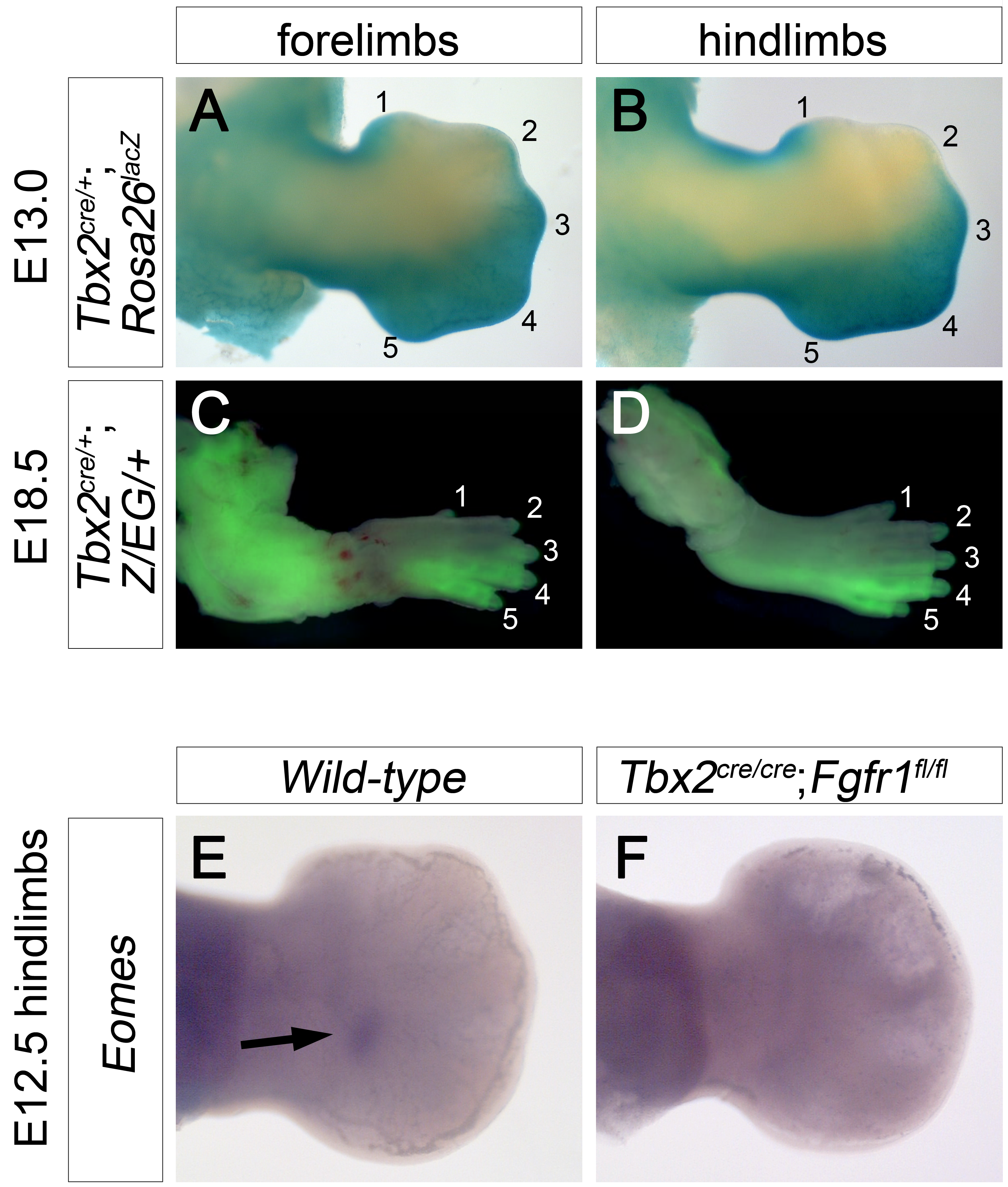

Supplement: Figure S2 — (A–D) Tbx2 expressing cells contribute to posterior digits 3, 4 and 5. (A, B) X-Gal stainings to detect ß-galactosidase activity in E13.0 Tbx2cre/+;R26lacZ/+ fore- and hindlimbs. Cells previously expressing Tbx2 as detected by β-galactosidase activity are present in the anterior and posterior flank mesenchyme and in the posterior half of the autopod encompassing the anlagen of digit 4 and 5, and partially of digit 3. (C, D) GFP-epifluorescence analysis in E18.5 Tbx2cre/+;ZEGGFP/+ embryos detects the final contribution of the Tbx2-cre + cell lineage to fore- and hindlimbs. Digit 3 is partially, digits 4 and 5 are completely derived from Tbx2 expressing cells. Note that the Tbx2-positive domains in the anterior and posterior flank mesenchyme do not substantially contribute to the E18.5 limb since they are most likely removed by apoptosis during development. (E, F) In situ hybridization analysis of Eomes expression as marker for digit 4 identity. At E12.5 wild-type hindlimb buds show a proximal domain of Eomes expression (arrow) that indicates formation of digit 4. Absence of signal in Tbx2cre/cre;Fgfr1fl/fl hindlimbs argued for a specific loss of digit 4 in oligodactic individuals. (TIF) [file pgen.1003467.s002.tif]

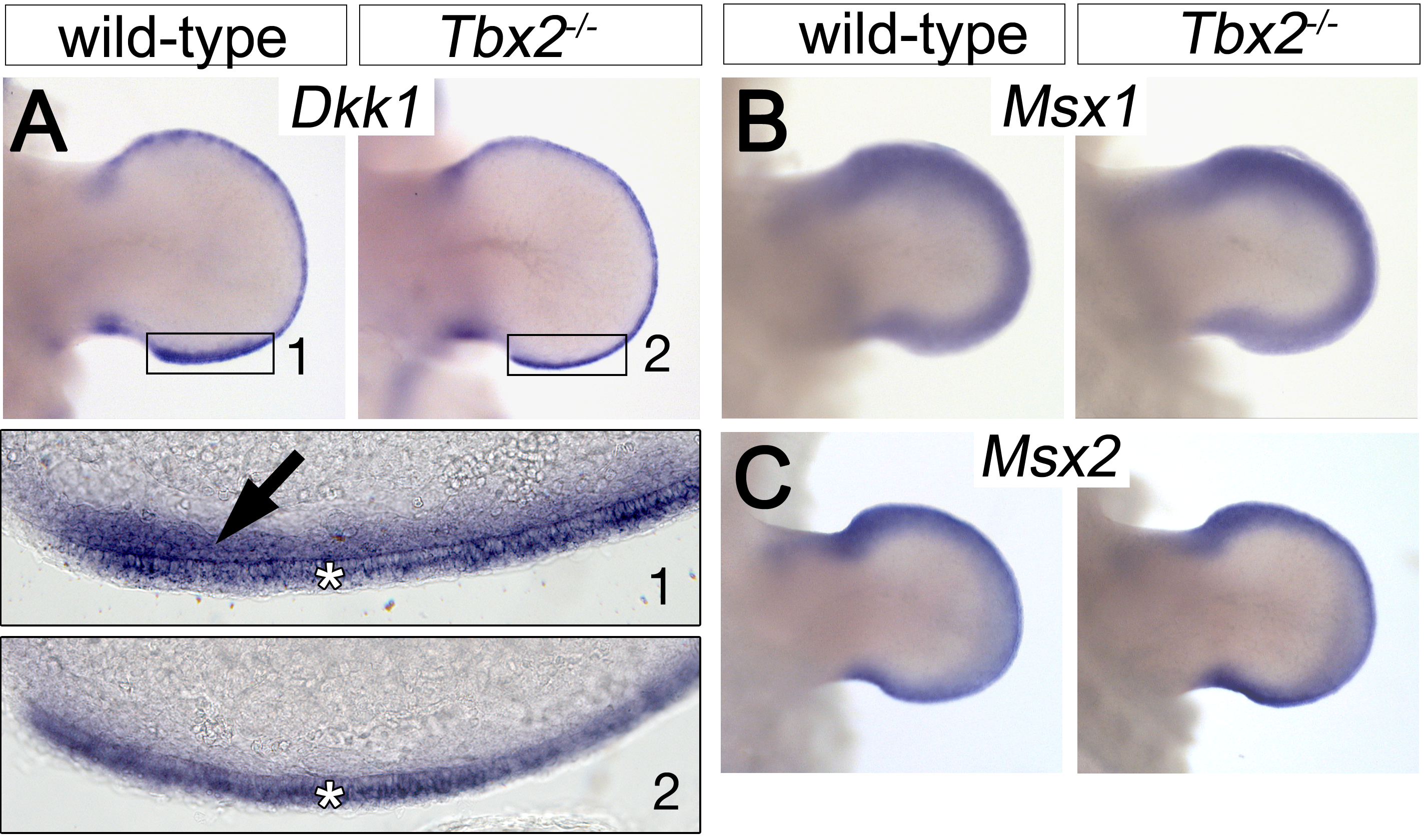

Supplement: Figure S3 — (A–C) Expression analysis of the Bmp target genes Dkk1, Msx1 and Msx2 by whole mount in situ hybridization in E11.5 wild-type and Tbx2−/− hindlimbs. Magnified regions (1 and 2) in (A) show loss of mesenchymal Dkk1 expression in the posterior limb bud region (arrow) of Tbx2−/− mutants but maintained expression in the adjacent AER (asterisks). (B–C) Unaffected expression of Msx1 and Msx2. (TIF) [file pgen.1003467.s003.tif]

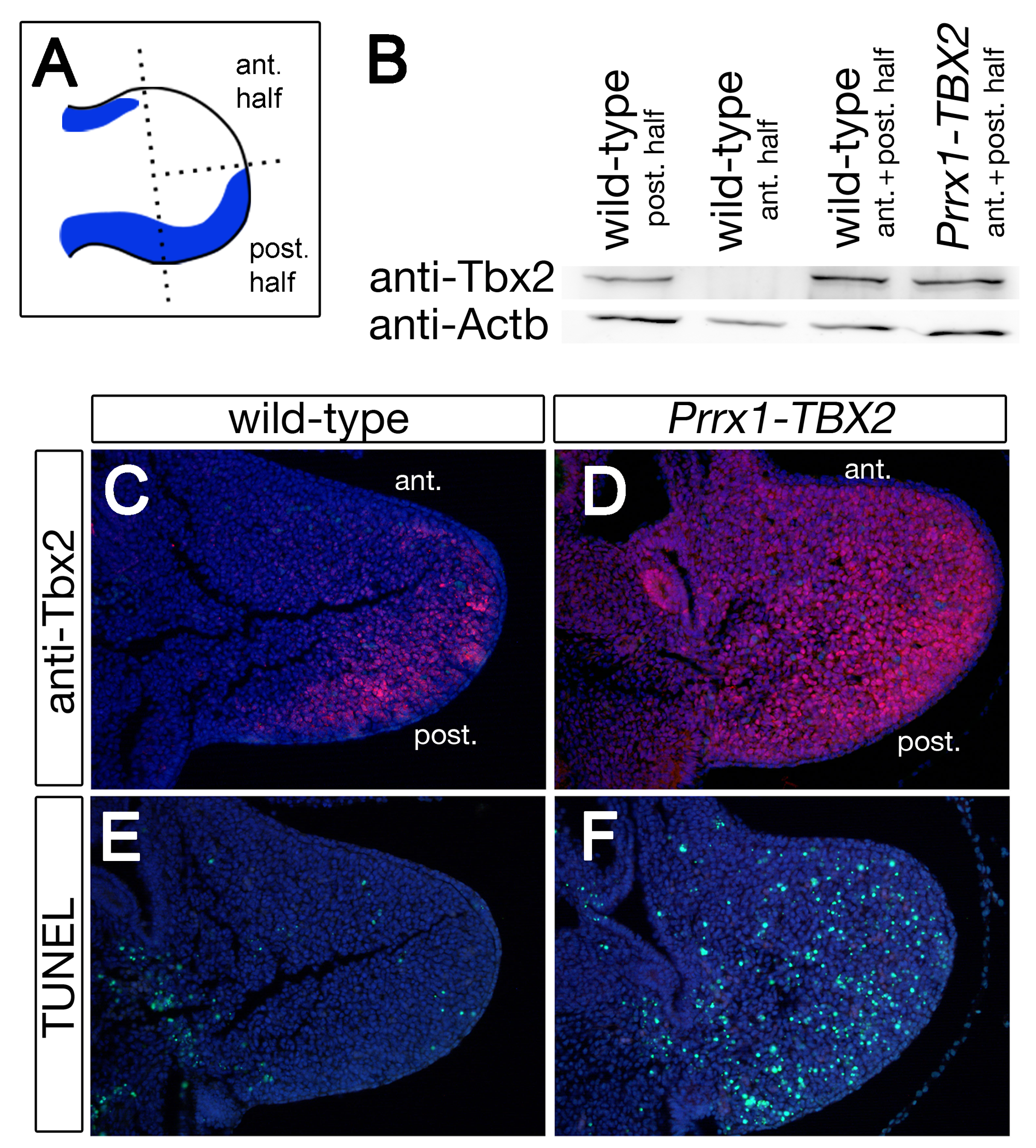

Supplement: Figure S4 — TBX2 protein levels and apoptosis in misexpression embryos. (A, B) Analysis of endogenous Tbx2 and transgenic TBX2 protein expression. Anterior and posterior halves of E11.5 forelimbs were collected (as shown in the scheme in A), and lysates were analyzed by Western blot. TBX2 misexpression in Prrx1-TBX2 (Prrx1-cre/+;HprtTBX2/Y) embryos was found at physiological levels. β-actin Western blot is shown as a loading control. (C, D) Immunostaining of endogenous and ectopic Tbx2 expression (red signal) in control and Prrx1-TBX2 embryos. Sagittal E10.5 forelimb sections are shown. Posterior restriction of Tbx2 in the wild-type (C) and ubiquitous mesenchymal expression of transgenic TBX2 protein (D). Same Tbx2 antiserum used as in (B). (E, F) TUNEL staining on sagittal E10.5 forelimb sections shows widespread mesenchymal apoptosis in Prrx1-TBX2 embryos. (TIF) [file pgen.1003467.s004.tif]

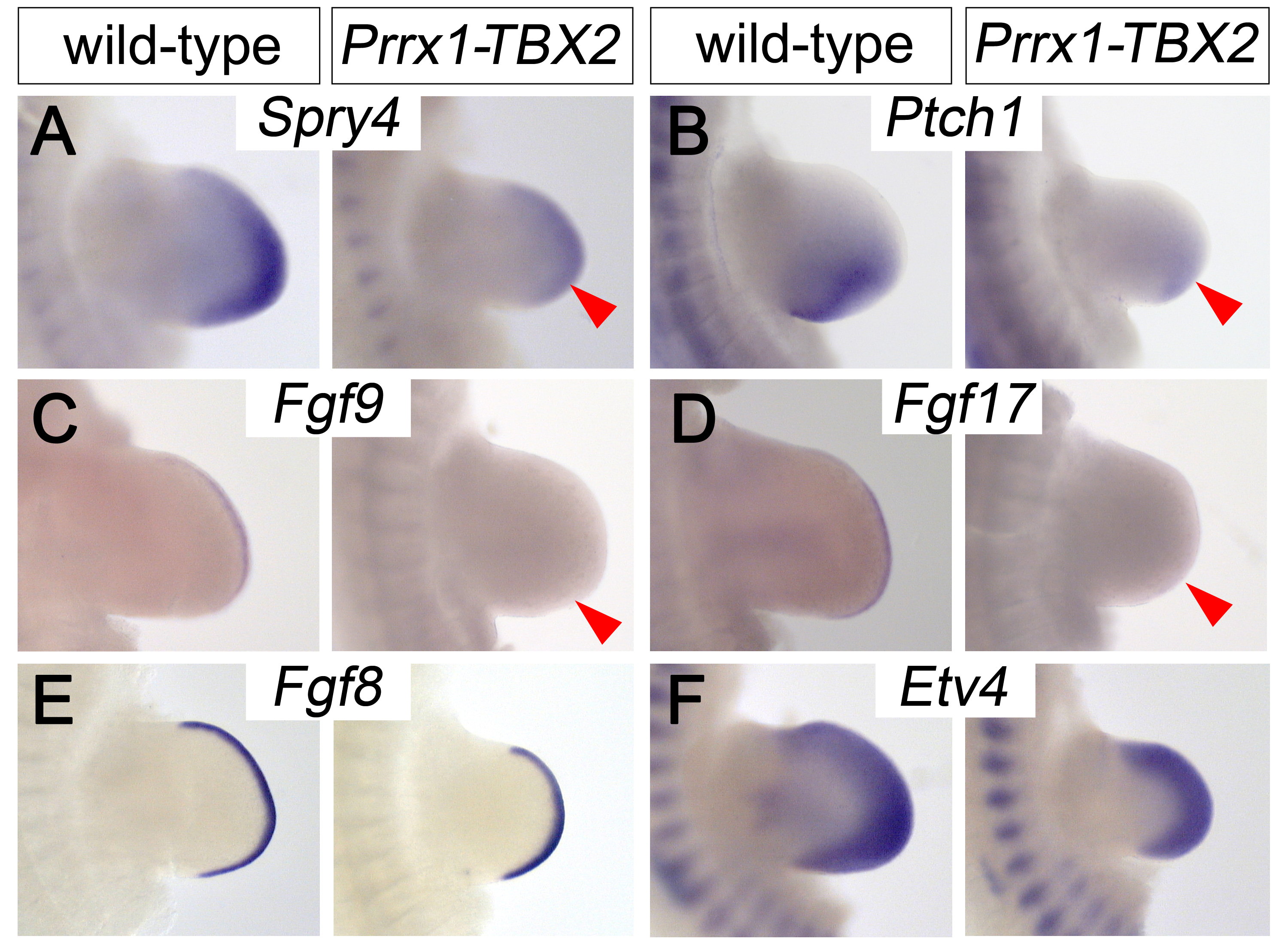

Supplement: Figure S5 — Selective disruption of posterior e-m signaling following TBX2 misexpression in the limb. (A–F) In situ hybridization analysis of Spry4, Ptch1, Fgf9, Fgf17, Fgf8 and Etv4 expression in E10.5 wild-type and Prrx1-TBX2 (Prrx1-cre/+;HprtTBX2/Y) forelimbs. Spry4, Ptch1, Fgf9 and Fgf17 are strongly reduced (red arrowheads) following TBX2 misexpression, whereas the levels of Fgf8 and Etv4 are not strongly affected. Note that in wild-type limb buds the expression of Spry4 is more restricted to the posterior-distal mesenchyme as compared to Etv4 that is expressed beneath the entire AER. (TIF) [file pgen.1003467.s005.tif]
